# Agile cyber defense: Enhancing digital substation resilience with SDN-based smart switching

**DOI:** 10.1371/journal.pone.0330521

**Published:** 2025-11-18

**Authors:** Sultan H. Almotiri

**Affiliations:** Department of Cybersecurity, College of Computing, Umm Al-Qura University, Makkah, Saudi Arabia; IGDTUW: Indira Gandhi Delhi Technical University for Women, INDIA

## Abstract

This paper provides an evaluation methodology and prototyped and tested a virtual Intelligent Electronic Device (vIED) for digital substations in a Real Time (RT) simulation. Based on the IEC 61850 standard and virtualization technology, the proposed framework aims to enhance protection, automation, and control systems for application in today’s substations. A new and very specific method of testing was conceived and used as an instrument for verifying the given framework and its potential based on different methods of communications, scalability, and functionality options. The analysis also examines the effectiveness of the SDCommNet for vIED frameworks about operational network re-scaling capacity, besides testing the impact of nominal and transient data traffic on the global performance of the system. Empirical findings demonstrate that the vIED framework always meets essential system characteristics, such as response time and network transfer latency, irrespective of configurations. Such findings act as a perfect foundation for future development of design and testing protocols for the virtualization of substation systems.

## 1 Introduction

With the growing complexity of modern electrical power systems, there is an emerging paradigm of digital substations, which are key to the reliable, self-intelligent operation of the grid [12,13]. Real-time communication and intelligent devices are heavily relied on these substations for control, protection, and monitoring functions. Because the industry is increasingly requiring technologies such as IEC 61850 to define the communication between Intelligent Electronic Devices (IEDs), new challenges are being faced at system scalability, cybersecurity, latency sensitivity, and operational flexibility [17]. Compared to traditional substations, which depend on fixed-function hardware, the requirements in terms of dynamic operations and resource constraints are extremely challenging to meet because of the rigid, constrained architectures of conventional substations. Virtualization technologies and Software Defined Networking (SDN) have enabled new transformational pathways of making substations agile, programmable, and resilient infrastructures [10,11]. However, integrating advanced technologies into time-critical domains, such as substations, presents its own set of complexities, including reliability, secure communication, and security in the face of both faults and cyber-attacks. This implies that the research on obtaining a balance between flexibility, resilience, and deterministic performance for digital substations has become an important research area [7,8].

There are some services in digital substations IEC 61850 provides in the foundation, such as GOOSE (Generic Object-Oriented Substation Events), Sampled Values (SV), and Manufacturing Message Specification (MMS) [9,11]. They are protocols that support the protection, relaying, fault detection, and automated switching applications that require low latency. Indeed, despite IEC 61850, the current substation architectures are still deemed vulnerable because they depend on static configurations and physically dispersed devices. Furthermore, communication and control logic in such systems are tightly coupled and cascading failures are prone to occur, as well as restricting their capacity for response to anomalies or cyber incidents. Substations are continuously digitalized, and the boundaries between the IT and OT domains have become blurry, and as a result, these infrastructures are susceptible to various types of cybersecurity threats [1]. Such protocol vulnerabilities could be exploited, forged packets injected, arbitrary time synchronization disrupted, or misoperation of circuit breakers done purposefully, with the possible effect of destabilizing and threatening grid stability or safety [5,6]. Thus, substations must have more architectural flexibility and stronger defenses that can adapt to changing threats in a dynamically survivable fashion to maintain continuity of operation.

These challenges are addressed by the virtualization of IED functionality from fixed hardware so that intelligent devices can be created in the form of Virtual IEDs (vIEDs) that can be scaled and replicated as well as rapidly deployed [3,4]. It can instantiate these vIEDs in virtual machines (VMs) or containers to adopt high availability, migration, and resource optimization features. Combining vIEDs with SDN that separates the control and data planes of the network allows programmable controllers to orchestrate, adaptively manage communication flows, prioritize traffic, and reconfigure the topology due to faults or attacks [2]. SDN provides unprecedented visibility and network control to logically control traffic flow in real-time, reroute traffic, and isolate malicious flows. In the substation context, these capabilities can significantly enhance the resilience and fault tolerance while maintaining protection scheme timing requirements that depend on IEC 61850 [14,18]. However, both vIEDs and SDN present an attractive potential, yet their combined deployment into the substations has only been minimally explored so far, especially regarding the performance evaluation for real grid events, fault scenarios, and cyber threats.

**Fig 1:**
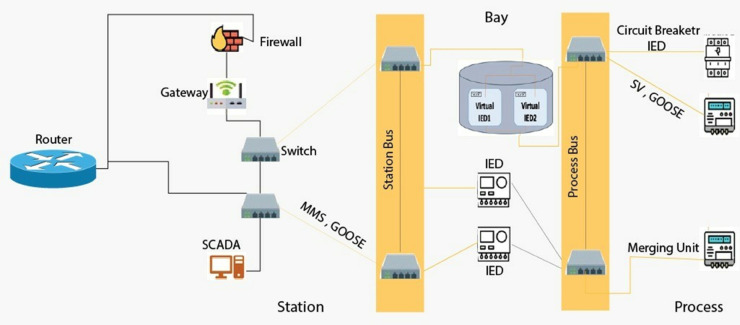
IEC 61850 substation communication architecture defined by software.

Then this paper puts forward an agile cyber defense framework of digital substations, Fig 1, based on SDN-enabled smart switching and virtualized IEDs for improving resilience, security, and flexibility. Our objective is to develop a complete solution that retains the advantages of digital substations while addressing their shortcomings, particularly in terms of high communication adaptability and low fault reaction. We use a hybrid testbed where RTDS and RSCAD [16] are used for real-time digital simulation and network emulation is done using Mininet and containerized vIEDs to run the tests in realistic power grid scenarios [20]. It supports GOOSE, MMS, and SV protocols and complies with IEC 61850 communication standards [15]. It offers flow prioritization, dynamic topology adaptation, real-time communication failure recovery, through logic that is programmable in SDN controller. The vIEDs are designed to behave as traditional IEDs, however, they behave in a virtualized, scalable environment. We successfully validate that under stress conditions, our system rejects stress while maintaining the same good properties of fault-clearing response times and communication determinism as in our baseline case [22]. We also test the system in response to several loads, including network loads, DoS-like attacks, and increased topologies.

This work explores the study of modernizing power system protection and control research, introducing a practical and experimentally valid architecture that bridges the modularity of virtualization and the agility of SDN. Compared to prior work using SDN or vIEDs applied in isolation to substation settings, our solution is a hybrid and encompassing solution for overall testing under operational dynamics. The key contributions of this paper include: (1) the design and implementation of a virtualization-based IED framework that supports dynamic instantiation and scaling of substation functions; (2) integration of an SDN-based controller with real-time flow control and fault recovery logic, designed specifically for substation-grade GOOSE messaging; (3) development of a hybrid evaluation platform incorporating RTDS, Mininet, and containerized environments to enable reproducible testing; (4) empirical validation of system resilience, latency response, throughput, and jitter metrics under both steady-state and failure-induced scenarios; and (5) an exploration of real-world implementation challenges, cybersecurity implications, and potential pathways for future work including redundancy schemes and integration Using AI-based detecting systems.

## 2 Related work

Significant research efforts have been made to enhance the flexibility, reliability, and cybersecurity status of digital substations throughout their evolution. Because of the great adoption of IEC61850 standards, most of the research efforts focused on the enhancement of communication efficiency and the completeness of the interoperability between intelligent electronic devices (IEDs) [18,23]. There is, however, a stream of literature which focuses on deterministic delivery of time-critical messages, such as GOOSE and Sampled Values (SV), which are important in protection as well as control schemes [21,24]. For example, studies have been proposed to enhance LAN architectures as well as message prioritization techniques to reduce delay and jitter in IEC 61850-based communication [25]. Despite the progress made by these approaches for enhancing real-time message delivery, they are limited in running on inflexible, unchangeable equipment and, beyond that, cannot react to network anomalies or to adaptively face the threats that emerge. As a result of the inherent rigidity of traditional substation topologies, static communication configurations and some other reasons, systems are prone to performance degradation and targeted cyber-attacks [26].

The second major body of work considers the integration of Software-Defined Networking (SDN) for the solution of these problems. SDN separates the control and data planes of a network and lets them to be programmable, centrally, and monitored in real time. Examples of researchers who used SDN to demonstrate how IEC 61850-based networks can benefit from flexible routing, topology reconfiguration, and rapid isolation of compromised nodes can be found [27]. In substation settings, it has been proven effective to use SDN to mitigate the impact of DoS and flooding attacks [28] through leveraging the dynamics of flow table updating according to traffic anomalies. Also, SDN is studied for traffic load balancing optimization, message congestion reduction and multicast GOOSE traffic management [29]. Although these advances were made, the majority of existing works execute SDN configurations in staticity or conduct traffic patterns emulation in the absence of traffic, thus, embodiments are lacking in real substation conditions within actual substation scenarios where they have embedded traffic into real IEDs, followed by power event simulation [30]. It leads to an unfavorable gap between theoretical grid resilience and its actual feasibility in live power grid conditions.

Another related stream of research is the area of virtualization in substation systems, and one of the specific aspects of virtualization is via the concept of Virtual IEDs (vIEDs). Virtualization aims at improving modularity, scale, and cost effectiveness of the system by separating protection and control functions from physical hardware. vIEDs projects such as the “Virtual Substation” initiative, Mondragon et al. [31] have shown that the behavior of physical IEDs can be accomplished through virtual machines or containerized environments. Finally, these works have evaluated the performance of vIEDs using synthetic datasets or through limited-scale hardware-in-the-loop simulations. Virtualization enables dynamic allocation of resources, managing redundancy and smooth deployment of new functionalities, but poses timing determinism, real-time processing and security assurance problems. Also, to the best of our knowledge, few studies have been developed to support the communication complexities that occur when virtual devices communicate through IEC 61850 protocols within the high-speed protection system [19,32]. Although some papers have investigated IEC 61850 virtualization during normal load conditions, there is no set of studies performing virtualization of vIED architecture using dynamic network reconfiguration strategies under grid faults and cyber incident scenarios.

Though the coexistence of SDN and IEC 61850 communication has been studied in several ways, only a few have focused on the combination with real-time protection schemes and virtualization. For instance, Karthika et al. [33] simulated that GOOSE traffic can be subject to a cyberattack in Mininet and presented how SDN can re-redirect packets during reconfiguration on the controllers. Sheikh et al. [34] also implemented an SDN-based protection framework on OpenDaylight, where they concentrated on route convergence speed. The packet latency or loss studies described here were run almost exclusively at the network layer and did not integrate or virtualize IED behavior [35,36]. As a result, these frameworks are yet to be practically applicable. Additionally, there hasn’t been a deep exploration of the effect of protection algorithms’ behavior in networks on the hand of SDN-controlled networks within virtualized environments. This is important because digital substation operation depends on the interoperability between real-time protective actions and underlying communication infrastructure [37]. Consequently, the contribution of such ideas in various aspects, without validation in real-time digital simulators like RTDS, is usually limited to the non-critical parts or just a concept [38].

**Fig 2:**
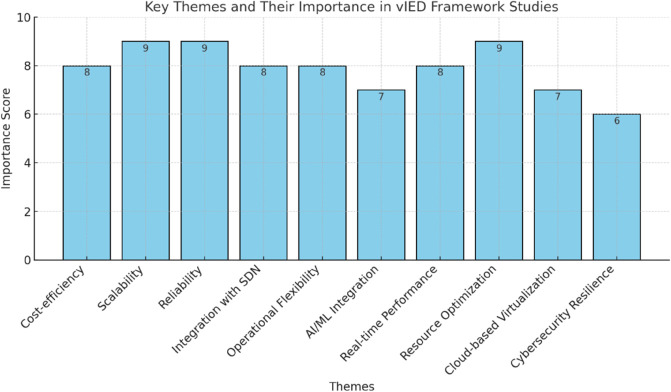
Key themes and their importance in vIED framework studies.

To date, there is a dearth of research that takes such a global, end-to-end view of digital substation resilience that balances programmable intelligence of SDN, modularity of virtualization, with communication demands of IEC 61850 Fig 2. The evaluation of these components in isolation has been explored, but to date, their joint evaluation in a real time, fault-injected, and adversarial environment remains popularised in the literature. Furthermore, there are neither many frameworks that stress test the interaction between cyber and physical layers under such conditions as in the case of network failures, high traffic load and/or targeted attacks on GOOSE or MMS messages [18,39]. In addition, most current testbeds are either strictly virtual, making them inapplicable to real substations, or heavily rely on hardware, hindering them in scalability and reproducibility for research [40].

In this regard, we fill the research gaps with a hybrid, SDN based architecture of a substation communication network utilizing virtualized IEDs and study its related behavior in realistic scenarios using a co-simulation testbed. Compared with previous studies, our work avoids the gap between abstract SDN control and real-world IEC 61850–based protection workflows by integrating virtualized logic vIED into real-time power system events through the RTDS. We stress both normal and fault cases and introduce stress testing for latency, jitter, throughput, packet loss, and recovery time for dynamic topologies. We also develop cyberattack conditions (link failures and denial of service) to measure the resilience of the SDN controller regarding its ability to preserve the protection signal performance. Beyond the architectural integration, the novelty of our approach lies also in its methodological strength, namely, we justify the equivalence between traditional IED and vIED performance, we deterministically determine the behaviour under IEC 61850 protocols and we propose a scalable design barely able to support future cybersecurity angularities like intrusion detection based on anomaly’s or attrition in blocks.

Finally, while there have been many studies on IEC 61850 communication, SDN control, or virtualisation separately, we present one of the few works that synthesise these into a coherent experimentally verified scheme of agile cyber defence in digital substations. We contribute a novel, practical and reproducible approach to building resilient, secure and flexible substation environments by demonstrating that virtualized protection logic is feasible in real-time and that SDN can manage, isolate and reroute critical communication paths involving GOOSE while ensuring GOOSE performance.

## 3 Methodology

To validate the IEC 61850-based virtual Intelligent Electronic Device (vIED), and evaluate a software-defined communication network performance, a robust methodology is developed and implemented in a controlled and scalable test environment. The methodology Fig 5 involves designing two interrelated systems: The functionality of the vIED framework is validated using a virtual substation simulation on an IEEE 5-bus power grid model, and the performance and scalability of the vIED framework is assessed on a Mininet-based emulated communication network under a range of network conditions. Below, each component is described in detail to ensure a full understanding of the process.

**Fig 3:**
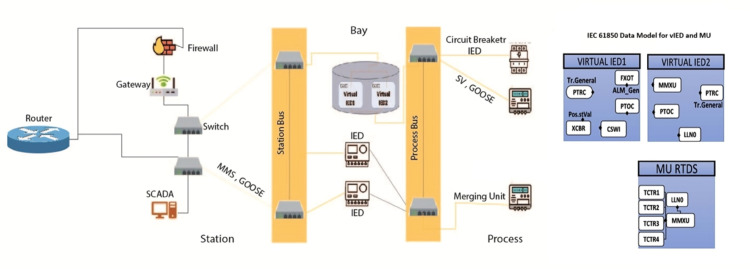
Proposed model.

The Fig 3 schematic design features a firewall at its center while protecting all internal network assets. The network consists of two main sections, which are named Gateway and Switch Bus. The critical infrastructure includes a router that handles network traffic between different networks and a supervisory control and data acquisition system as well and a station for central control functions. The Switch Bus section contains numerous devices named SMA, ranging from SMA5 to SMA599 and possibly includes functional units or subsystems denoted by GOODS in the list. In this organization-scale network, k an established device-naming protocol, seems to exist because the numbering system displays regular repetition patterns with intermittent double entries (such as SMA59,2, which appears two times). Application of network traffic controls through the firewall enables security safeguards for critical system Gateway traffic passing between it and distributed devices, Switch Bus, while preserving network connectivity. The network structure illustrates how the firewall serves as a central checkpoint to secure Gateway assets, protecting them against threats originating from the wide Switch Bus domain.

### 3.1 vIEDs and SDN controller logic

A modular design is justified with the implementation of the proposed SDN-enabled digital substation architecture based on a scalability and fault-tolerant operation of vIEDs and programmable communication logic in Firmware via Software Defined Networking. In order to emulate real substation behavior, we developed a containerized framework for vIEDs replicating the behavior of the physical IEDs in terms of parsed data, message transmission and inter-device interactions. A lightweight Docker container instantiating each vIED with a custom service built onto an IEC 61850 compatible library (in this case, libIEC61850) is instantiated. In particular, this library is the core communication stack within each vIED for the encoding, decoding and state machine management of GOOSE, Sampled Value (SV) and Manufacturing Message Specification (MMS) messages. The IEC 61850 specification defines the protocols over the UDP and TCP transport layers, SV and GOOSE use multicast Ethernet frames for fast delivery and redundancy. In a logical node model specified from a standard Substation Configuration Description (SCD) file through the use of the Substation Configuration Language (SCL), an XML schema that translates physical device functions into physical devices, logical devices, document nodes (LNs), data objects, and attributes, the vIEDs interpret messages. The vIED processes its fragment of the SCD configuration and generates, at runtime, control logic and the associated data model for communication through a data-driven binding.

The internal architecture of a vIED is made up of several software threads that are merged into an extensible publish-subscribe model. SV subscription and decoding are handled by one thread, GOOSE message publication and event-based decision making are carried out by the other, and the third provides an interface with central control center (CC) using MMS interface for status reporting and command execution. Streams from merging units (MUs) are merged, are decoded, and sampled phasor data and this information are supplied to the SV thread that triggers local logic based on trip thresholds or overcurrent detection. IEC 61869-9 data schema is used to parse each stream and payload elements are induced in structured memory buffers (mapped to I.E.C. 61850 data object names). This might, for example, be used to trigger state changes of the LN’s PTOC (Protection Time Overcurrent) or PTRC (Protection Trip Conditioning) when the rising edge or threshold exceedance is seen within the sampled values. When a trip condition occurs, then it creates a GOOSE message with a specific dataset ID and sequence counter. This type 1A message is encoded in this multicast Ethernet channel using a fixed Application ID (APPID), destination MAC address and time to live counter according to the strict time constraint ≤ 3ms end-to-end time for protection action. The synchronization is performed using the Precision Time Protocol (PTP) and data is communicated within Redundant Forwarding Paths (RFP) which are provisioned based on SDN routing logic.

SCD files are used since each vIED can always maintain the full understanding of its logical role in the substation topology. When initializing the vIED, the SCL parser implemented in Python is used to parse the XML structure of the SCD file. The logical devices are extracted by this parser, mapped to data models and an object tree is initialized for a device-specific object tree following IEC 61850/7-3/7-4 standards. The parser creates object classes for each logical node corresponding to such function blocks as XCBR (circuit breaker), CSWI (switch control, or FXOT (alarm signal), and binds them to communication service via GOOSE control blocks and datasets. Internal timers and condition monitors that act as event triggers are used to register these object classes. The implementation of the vIED state machine consists of a finite state automaton (FSA) whose transitions are initiated by SV inputs or received GOOSE messages. The state transitions function uses a cyclic executive loop design that polls inputs every millisecond to meet real-time requirements of IEC 61850 timing standards.

SDN stands as the key element to direct traffic flows between the process and station buses from a communication perspective. Mininet serves as the platform to deliver SDN network implementation, which models an actual Ethernet switching fabric, while OpenFlow 1.3 supports each switch in the topology. The controller operated through the Ryu framework connects to OpenFlow switches by TCP over the southbound interface. Python programming enables the controller to maintaina global network view information that includes topology data alongside port states and MAC address tables and flow statistics. Every Ethernet frame, such as GOOSE or SV packets, that reaches an OpenFlow switch, for instance, SW1, undergoes a local flow table match process. The absence of matching rules activates the SDN controller to receive the packet header through the *packet*_*i*_*n* event. Upon receiving the packet, the controller operates its flow decision logic for processing. Our design includes a reactive flow installation mechanism that uses Ethernet type, together with VLAN ID, along with APPID for GOOSE and multicast destination MAC as classification criteria. The controller determines the packet’s disposition to send it to a GOOSE subscriber or drop it or redirect it through an alternate link because of detected failures based on these conditions.

The SDN controller applies flow allocation through an algorithm that bases decisions on network utilization, together with interface status and defined traffic priorities. The controller utilizes OpenFlow meter tables with QoS policies to tag and queue GOOSE and SV traffic classes through assigned VLAN IDs. A hash map controlled by the controller functions as a data structure that stores precomputed Dijkstra shortest-path output ports based on (source MAC, destination MAC, EtherType, VLAN ID) tuples. When link or switch failures happen, the controller receives topology updates through LLDP, resulting in an immediate recalculation of shortest path trees. The controller executes *flow*_*m*_*od* message distribution to affected switches for delivering time-sensitive packet traffic uninterrupted. A clustered high-availability setup with primary-backup architecture supports the SDN controller in order to prevent it from becoming a single point of failure. The control nodes communicate heartbeat messages to their peers while using leader election protocols to select their control authority when failures occur.

**Fig 4:**
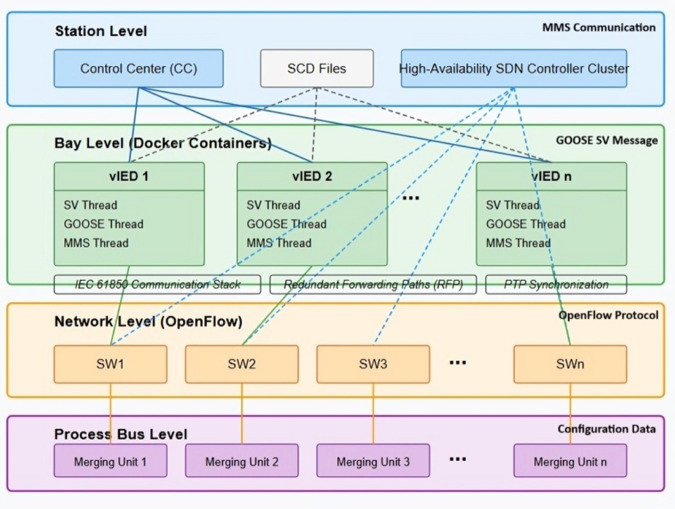
SDN-enabled digital substation architecture.

The hybrid system’s Fig 4 behavior required testing through scenarios that included sudden traffic shifts together with link outages and GOOSE network floods. The OpenFlow *stats*_*r*_*equest* API enabled the SDN controller to obtain network statistics regarding port traffic yet the controller evaluated its routing performance using a timing-based approach for delay injection tests. The vIEDs were observed for their performance in processing SV streams while sending GOOSE messages during the required 4 ms fault-clearing period. The DNS logic, together with VLAN-based traffic segregation and pre-tagged priority queues managed to sustain process bus determinism during times of intense traffic conditions. The designed solution enables expansion from three to twenty vIEDs while preserving both SV and GOOSE operation, which proves the ability to implement large-scale virtualized substations with SDN capabilities.

**Algorithm 1 Handling Packet_In event and fault monitoring in SDN controller.**

1: **procedure** Packet_in(packet)

2:   header ← extract_headers(packet)

3:   eth_type ← header.eth_type

4:   vlan_id ← header.vlan_id

5:   src_mac ← header.src_mac

6:   dst_mac ← header.dst_mac

7:   **if** eth_type = GOOSE_TYPE **and** vlan_id = GOOSE_PRIORITY **then**

8:    **if** dst_mac ∈ active_topology **then**

9:     path ← compute_shortest_path(src_mac, dst_mac)

10:     **for all** switch ∈ path **do**

11:      match ← {in_port, eth_type, vlan_id, src_mac,

dst_mac}

12:      action ← forward_to(next_hop_port)

13:      install_flow_rule(switch, match, action)

14:     **end for**

15:    **else**

16:     log("Destination unreachable: dropping GOOSE packet")

17:     drop(packet)

18:    **end if**

19:   **else**

20:    process_standard_traffic(packet)

21:   **end if**

22: **end procedure**

23:

24: **procedure** Monitor_Topology

25:   **while** true **do**

26:    **if** detect_link_failure(link) **then**

27:     affected_flows ← identify_flows_using(link)

28:     **for all** flow ∈ affected_flows **do**

29:      src ← flow.src_mac

30:      dst ← flow.dst_mac

31:      new_path ← compute_alternate_path(src, dst)

32:      remove_old_flow_entries(flow)

33:      **for all** switch ∈ new_path **do**

34:       match ← flow.match

35:       action ← forward_to(next_hop_port)

36:       install_flow_rule(switch, match, action)

37:      **end for**

38:     **end for**

39:    **end if**

40:   **end while**

41: **end procedure**

The described Algorithm 1 demonstrates how SDN controllers react to IEC 61850 GOOSE messages based on their requirements. The forwarding operation comes first by VLAN and EtherType matching and creates dynamic per-hop flow rules. When a topology failure occurs, the controller calculates alternative transit routes while instructing flow tables for maintaining uninterrupted critical protection data delivery.

### 3.2 Justification of design choices

This work selected particular tools and frameworks and design approaches to achieve both realistic behavior and flexible functionality, together with scalable performance and standardized substation communication protocol compatibility. Each component selects attributes that fulfill the requirements of digital substations following IEC 61850 specifications as well as the ability to run experiments in normal operational and adversarial situations. These choices are founded on technical functions but also ensure the future capabilities of reproducing experiments and operating between diverse deployment environments.

The Ryu SDN controller was selected due to its open-source status, extensive development community, and capability to manage and control flow rules through OpenFlow 1.3 at a packet-granularity level in real time. Teachers and students can easily add new custom modules to the Ryu system because its core runs on the Python programming language. Ryu stands out from OpenDaylight and its Java configuration due to its lightweight runtime that Python-based APIs make extendable and allowing for fast implementation of reactive packet handling alongside topology discovery and application-layer security policies without excessive overhead. The modular system structure supported fast development during which it was easy to develop and debug new control protocols that redirected GOOSE and SV messages under different failure or attack situations. Ryu proved advantageous for time-sensitive communication infrastructure such as the IEC 61850 process bus since it offered effortless Mininet and REST API integration for flow rule orchestration.

The virtualization implementation consists of combining Docker containers and QEMU-KVM virtual machines through a dual-mode strategy. Containers running Docker were designated to host most virtual IEDs (vIEDs) since they provide lightweight operation, together with speedy container boot time and minimal resource utilization. The technology has ideal properties that make it suitable for testing bay-level devices in large test environments that need rapid IED replicating systems. Docker containers delivered functional consistency to developers because they bundled each virtualIED with its required components while establishing a secure boundary against the host environment, making deployment of GOOSE/SV services easy. QEMU/KVM-based VMs were included in our system, yet we also integrated them because they offered necessary compatibility with legacy systems as well as hardware-level simulation capabilities. The technology was designed to support complete operating system environments that performed old-fashioned IED architecture emulation by replicating hardware requirements instead of using containerization methods. Our framework evaluation included testing within both full-virtualization and lightweight virtualization environments by means of a hybrid virtualization design, which showcased flexibility for different deployment configurations.

Our decision led us to Mininet, as it was necessary for emulating the Ethernet switching fabric found in substations. Through Mininet users can create OpenFlow-enabled virtual networks which allow them to control switch bandwidth and link parameters, including delay and packet loss rates. Its cooperation with Ryu delivers the best framework for assessing real-time flow rule propagation while testing reactive routing logic and real-time traffic rerouting capabilities. Mininet allows developers to simulate critical network faults through its integrated fault injection features, which recreate switch and link failures as well as traffic surges vital for assuring SDN-based process bus resilience. The evaluators examined GNS3 and CORE yet selected against them because of their limited support for OpenFlow and absence of built-in Python functionality,y which would create challenges for automatic controller interaction.

The communication stack implements the IEC 61850 standard as its foundation because the standard represents the prevalent industrial choice for digital substation automation. Modeling realistic protection messages and analog measurements, as well as device control scenarios, became possible through its specific communication profiles, which included GOOSE and SV, and MMS. By using the standard, we can maintain operational compatibility between our framework and industrial equipment as well as software instruments that professionals implement in substations. LibIEC61850 offered us an open-source library platform that supports complete functionality for IEC 61850-8-1 and 9-2LE profile standards. The vIED logic could perform publisher-subscriber operations and dataset management while parsing SCL-based configuration files through this specific library. Real SCD files obtained from operational utility deployments provided our virtual devices with operational realism through realistic configurations that included logical devices in addition to nodes (PTOC, PTRC, CSWI) and dataset definitions.

The selection of these framework elements allows the framework to meet experimental flexibility requirements while delivering practical device likeness functionality. The research tools fulfill unique standards-based goals, which include live traffic programming and IED virtualization and simulation capabilities as well as network device reproduction and controlled malfunction simulation. The clear framework architecture enables expansive system validity checks as well as repeated platform utilization for conducting further research work, such as implementing intrusion detection and AI-based flow control components. The design process decisions represent a deliberate method to solve the specific requirements of secure agile substation automation.

### 3.3 Validation of the vIED framework

**Fig 5:**
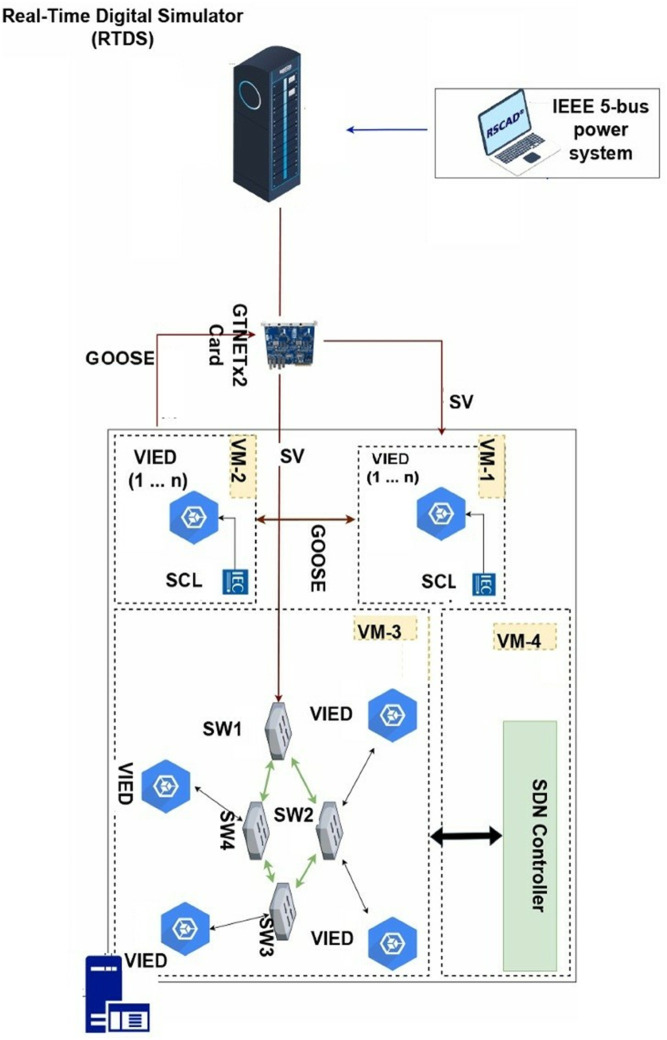
RTDS: a real-time digital simulator for simulating power systems.

In the context of the IEEE 5-bus power grid model, IEEE 5-bus power grid structure, the framework Fig 5 vIED (Virtual Intelligent Electronic Device) was validated using advanced simulation environments, i.e., the RTDS (Real-Time Digital Simulator) and the RSCAD (Real-Time Simulation and Control and Automation Design). Specifically, this power grid model was chosen because it efficiently models the behavior and interactions within a single, local digital substation. By designing and implementing this test environment, we were able to focus on validating these local communication protocols and interactions in the system, so that they could work in real life. The scalability of the vIED framework was also thoroughly investigated by zetetically decreasing the number of virtual IEDs and volume of data to be exchanged across the network. This allowed the system to scale as the degree of the network interaction increased, since the scale of the network increased. To pIEC-compliantmpliant messages central to power grid communication, an IEEE 5 bus model was configured with IEC 61850 compliant messaging. The messages contained Generic Object-Oriented Substation Event (GOOSE) messages or Manufacturing Message Specification (MMS) reports or Sampled Values (SV). Next, these were transmitted to internal IEDs simulated in the RTDS environment as well as external vIEDs indexed on dedicated virtual machines (VMs) that further enhanced the realism of the testing environment.

**Fig 6:**
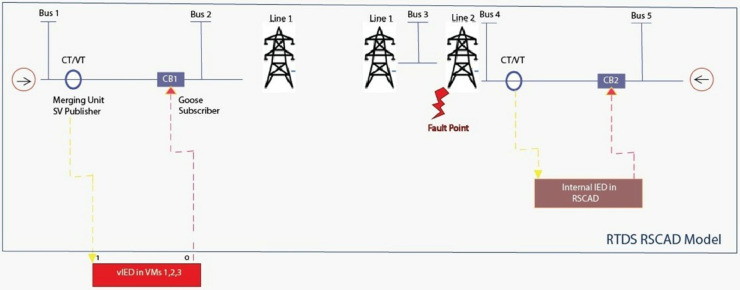
IEEE 5-bus system hybrid vIED testbed.

The protection and control mechanisms, Fig 6, commonly found in real-world power substations, were simulated in the model setup using two controllable circuit breakers, CB1 and CB2. These mechanisms are essential to the reliable operation of electrical grids since circuit breakers trip the flow of electricity in response to faults or otherwise abnormal conditions, as a means of protecting both equipment and staff. For the sake of comparing internal versus external control mechanisms within a digital substation environment, this simulation used Circuit Breaker 1 (CB1) and Circuit Breaker 2 (CB2) as two different methods of controlling circuit breakers.

1. Circuit Breaker 1 (CB1) and External vIED: A distributed vIED that was hosted on a physical server and connected to the RTDS subnetwork controlled CB1. Virtual Intelligent Electronic Device (or vIED) is a software-based emulation of traditional hardware IEDs (Intelligent Electronic Devices) found in substations. In this case, the external vIED was hosted on a physical server to simulate a true world scenario whereby the control and protection functions for circuit breakers could be located remotely, or from a central location, or another part of the power grid. The vIED interacts with the RTDS subnetwork, which models real time electrical grid behaviors and sends commands to CB1 to open or close based on different system conditions. The same setup is analogous to what might be used for remote control in a substation where the control of critical items such as circuit breakers could be exercised from a position that is distant from the coach itself. In this setup, the vIED messages use the IEC 61850 communication standards (GOOSE (Generic Object-Oriented Substation Event) messages) to communicate with CB1 and cause its operation in response to events in the simulation environment.
2. Circuit Breaker 2 (CB2) and Internal Protection IED: Whereas, the internal protection IED for CB2 was simulated directly within the RTDS environment itself. Physical or virtual device within the substation’s control system that can monitor substation’s parameters such as current, voltage, and frequency; detects faults and commands control devices, such as circuit breakers when needed, is an internal protection IED. The internal IED in CB2 was simulated in the RTDS environment so that it and the circuit breaker were part of the same simulation environment, as the external vIED setup for CB1, which was hosted on a separate physical server. System conditions were monitored in real time, and the control of CB2 could be performed by the internal IED that evaluated these conditions and directly drove CB2 control commands through predefined protection algorithms, e.g., overcurrent or undervoltage.
3. Dual Control Approach and Functional Equivalence: The functional equivalence of vIEDs and traditional hardware based IEDs was validated by using the dual control approach comprising both external vIED control for CB1 and internal IED control for CB2. This approach compared the virtual IED and physical IED control of a circuit breaker in similar conditions to determine the difference between the two. Researchers were able to simulate both setups in parallel to explore any discrepancies between the expected behavior of each type of device and decide on how the virtual system (vIED) can operate with the same reliability, speed and accuracy as a traditional hardware based IED. Of note, if researchers could compare how CB2 internal IED responded to system changes quickly and accurately versus the external vIED controlling CB1, it would help researchers understand the performance of this system. The purpose of the comparative analysis was to determine whether the virtual IED could achieve the same operational outcomes (such as detecting the faults) and command the appropriate protection action (i.e., opening the circuit breaker) with the same precision and reliability as the internal protection IED.

The major goal of this was to confirm that the vIED framework would or exceed the performance and reliability goals of conventional, hardware IEDs, specifically for control and prevention of important electric power grid elements such as circuit breakers. That’s a practical way of saying that in practice, the vIED system needs to be able to react quickly to faults in the system, interpret data correctly, and send the right command to the circuit breakers to keep up the stability and safety of the power grid.

Using the dual control setup, researchers then tested not only the vIED framework functionality but also its robustness with respect to fault detection, protection response times and communication reliability. This testing is essential because our goal is for vIEDs to be what is essentially a replacement for traditional IEDs while still being as safe, reliable and effective as possible. As power grid management increasingly relies on virtual devices execution in a modern substation environment where real-time protection and control is critical, the ability to operate both traditional and virtual devices seamlessly in the same environment is essential for digital substations adoption and the wider adoption of virtual devices in power grid management.

The use of a merging unit (MU) in the RTDS RSCAD environment was another fundamental element of the testing. It is the merging unit, that first published voltage and current measurements from a certain bus in the IEEE 5-bus model in IEC 61850 Sampled Values (SV) format. Merging unit significance was based on its function in delivering real-time data streams, strictly following the IEC 61850 standard, which is absolutely necessary for ensuring information integrity and accuracy in digital substation environment. Integral to evaluating how well the vIEDs can process high frequency data streams and perform protection and control tasks in real time were these sampled values. Processing of these data streams precisely is crucial for monitoring grid performance and a prompt response to any abnormal condition that may occur.

An additional implementation of a GOOSE (Generic Object-Oriented Substation Event) communication interface was later added to the IEEE 5-bus model. This interface was a critical step to validate since it subscribed to electronic trip signals coming from the exterior vIEDs. With this setup, the communication system was able to validate the response of individual devices in the system as well as the bidirectional communication between the devices within the substation. The singular feature that was particularly useful to testing the efficacy of protection schemes was the vIED’s ability to send a trip signal to cause the action of circuit breakers when required for abnormal system conditions, like a fault or overload. Real time testing of the vIED framework under these events proved that the virtual devices can perform such events accurately and reliably, providing essential data to assess the robustness and reliability of the virtual devices when engaged in protective control activities.

The vIED framework was deployed and tested on the deployment and scalability axis through deployment of the external vIEDs on different virtual machines (VMs) on which each VM contained a single vIED. A real Substation Configuration Description (SCD) file offered by EDF R&D was used to configure these VMs, adding to the validity of the testing environment. It was then possible to evaluate the performance of the framework’s deployment of vIEDs across multiple VMs in various scenarios including different resource usage levels and network load levels. Within each VM, multiple containers were deployed to simulate the use of more resources and network traffic — they proved invaluable in pinpointing the scalability of the system. In order to assess the limits of data exchange and inter-vIED communication across a virtual network, we chose to deploy multiple VMs, rather than a single resource-heavy VM. Using this method, the communication performance of the system was evaluated under different conditions, experimenting with different configurations such as MMS reports and GOOSE control blocks. This allowed the validation team to see if the communication stack could handle increased data volume and network complexity without compromising performance or reliability which would mean that the system could scale.

The extensive testing of the vIED framework was critical to ensure that it will perform well in the actual time digital substation context, that it will be operational in actual world applications and that it can scale and communicate well in progressively much more complicated systems.

### 3.4 Performance validation of the software-defined communication network.

**Fig 7:**
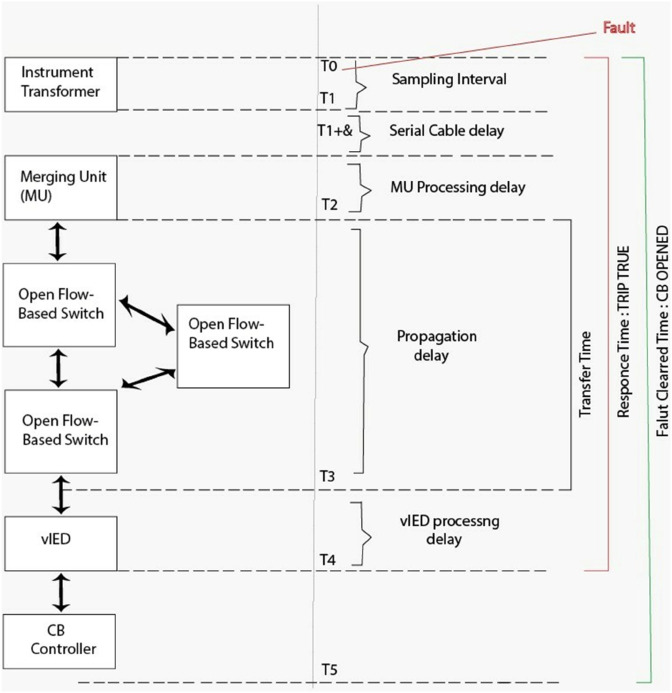
Real-time simulation: makes use of RTDS to simulate power system events and dynamics in a precise and lifelike manner.

To replicate the communication architecture Fig 7 of a substation at the process level and bay level, this study emulated a software defined communication network with the Mininet network simulator. We also carefully engineered the test environment to connect with the RTDS and GTNETx2 card exactly so that data that the RTDS injects into the RTDS socket with the GTNETx2 card can be sent to a virtual host in Mininet over socket bind. We configured the Mininet environment handling Sampled Values (SV) streams, which were multicast onto the Mininet network to emulate different communication scenarios with scaling factors. Mininet virtual hosts which played the role of virtual Intelligent Electronic Devices (vIEDs) received these SV streams representing real-time data from power system equipment such as voltage and current measurements. This configuration was aimed to validate end to end communication performance between several virtualized devices—these virtual devices were both real physical hardware, ones that run the IEC 61850 standards used in substations, as well as emulated ones; both end-to-end communicating with each other, receiving the data correctly, and processing it according to the IEC 61850 standards. By integrating Mininet with RTDS, the data flows that would occur in a modern digital substation were tested with realistic testing to guarantee the framework had the capability to deal with complex real-world communication scenarios.

In addition, the test setup included certain configurations in order to interact between Mininet virtual host through multicast communication. In a particular scenario, for example, host1 would subscribe to the SV streams being sent from the RTDS over socket communication with switch1. Host1 then published a Generic Object-Oriented Substation Event (GOOSE) message after receiving the SV streams. Host3 then received this message, connected to Switch3, so that it could propagate GOOSE messages to the network. Validating the robustness and efficiency of communication across the network required the flow of both SV and GOOSE messages. By establishing multicast communication setup, the system was able to deliver a thorough evaluation of the environment in which real-time data can be transmitted and received without loss of integrity or timeliness. That was a necessary step in order to evaluate the extent to which the system would be able to handle multiple communication protocols, in particular those that require time-sensitive data to be transferred between various substation devices, e.g. fault reports and control commands.

In this scalability testing the number of Mininet hosts was increased in the network to emulate more traffic load. To test scalability in terms of host count and spectral traffic volume, these new hosts published additional SV streams into the network, which the researchers used to evaluate how the framework would scale. This section of the testing was intended to stress the communication network to its breaking point, though there would be high traffic flow, potentially network congestion and latency. Scalability tests showed that the vIED framework was able to support ever more complex substation scenarios without sacrificing performance, which was important for large-scale deployments. Network conditions were tested out for the different operational scenarios such as an increase in network traffic, a variant topology of the network and increasing latency. In conjunction with these scenarios, they were able to simulate a broad range of real-world situations in which network performance may begin fluctuating (due to instances of heavy data loads or the necessity to reconfigure the network topology on the fly).

A combination of tools was used to monitor and analyze network performance, iperf3 and Wireshark. Key performance metrics such as latency, bandwidth and round-trip time have been obtained using the iperf3 tool under different network conditions. For example, this tool was particularly useful in observing how network events, such as link failures, network congestion, and so forth, disrupted overall communication performance. This facilitated the collection of detailed, quantitative data on the network to assess the network’s robustness as well as its capacity to continue maintaining high-quality communication. Packet-level data was captured in Wireshark to get a deeper insight into which communication flows were taking place among different devices. The researchers were able to use Wireshark and verify that the data sent over the network was properly transmitted according to IEC 61850 communication standards and did not get lost or corrupted in transmission. iperf3 and Wireshark helped to monitor the network performance fully, including both performance metrics and integrity of communication.

In addition, the study tested the adaptability of the software defined communication network on dynamic parameters introduced by the dynamic change of topology during testing. These actions, such as dropping network links, shutting down switches, and inflating traffic loads, would be taken to observe what would happen to this network when the shocks hit. We designed these tests so that network failures or topology changes would be unexpected in real-world conditions. The researchers observed how the network responded to these changes and used that to gauge the network’s resilience and its capability to maintain stable performance under challenging cases. To generate the network’s performance metrics visual representations the data gathered in these dynamic tests were processed with an iperf3 plotter and preprocessor tools. These visual tools both gave a clear understanding of how the system responded to different stressors and served as valuable tools to understand the system’s overall robustness and reliability.

The use of this systematic and thorough methodology allowed full and comprehensive validation of IEC 61850 based vIED framework and supporting software defined communication network. The study was able to provide a robust analysis of the functional and performance aspects of modern digital substation systems by integrating simulation, emulation and real time monitoring tools. This validation process provided critical insights into the performance capabilities of the vIED framework and evidence of its capability to operate in the demanding and complex environments of digital substations. The study set a solid foundation for future development in the field by validating the communication protocols as well as the performance of the overall system in the environment of advanced substation automation and control systems.

### 3.5 Results of validation tests

#### 3.5.1 vIEDS performance testing for real-time protection.

The vIED framework was deployed and tested on the deployment and scalability axis through deployment of the external vIEDs on different virtual machines (VMs) on which each VM contained a single vIED. A real Substation Configuration Description (SCD) file offered by EDF R&D was used to configure these VMs, adding to the validity of the testing environment. It was then possible to evaluate the performance of the framework’s deployment of vIEDs across multiple VMs in various scenarios, including different resource usage levels and network load levels. Within each VM, multiple containers were deployed to simulate the use of more resources and network traffic, which proved invaluable in pinpointing the scalability of the system. In order to assess the limits of data exchange and inter-vIED communication across a virtual network, we chose to deploy multiple VMs, rather than a single resource-heavy VM. Using this method, the communication performance of the system was evaluated under different conditions, experimenting with different configurations such as MMS reports and GOOSE control blocks. This allowed the validation team to see if the communication stack could handle increased data volume and network complexity without compromising performance or reliability, which would mean that the system could scale. The extensive testing of the vIED framework was critical to ensure that it will perform well in the actual time digital substation context, that it will be operational in actual world applications and that it can scale and communicate well in progressively more complicated systems.

This article focuses primarily on how we can measure the average total response and fault clearing time to validate the setup derived. For the baseline test cases, it is initially assumed that time dependent delays resulting from the overcurrent protection algorithm can be safely neglected and the transfer time is observed, rather than explicitly measured. Some additional time measurements (cyclic execution time and jitter) for future setups optimized for real time deterministic evaluation may be made. We classify three main criteria for evaluating vIED performance by assessing how quickly it can adapt to different network configurations, load changes or system reconfigurations. These are C1: Communication Protocol and Exchanged Data that tests different IEC 61850 data models and protocols for exchanging trip signals; C2: Scalability, which seems the number of concurrently running vIED with hybrid container and virtual machine (VM); C3: Functional Changes which measures the time delays in the overcurrent protection algorithm.

**Fig 8:**
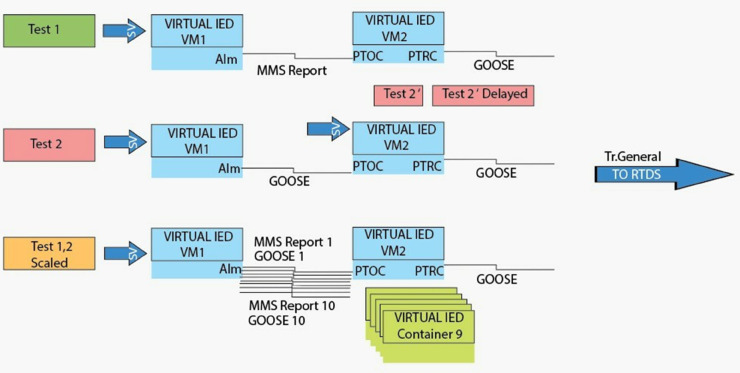
vIED testing scenarios. Three examples illustrate different IEC 61850 communication methods and scalable vIED deployment.

The vIED framework was validated in 6 test scenarios. Using a basic overcurrent protection algorithm, these scenarios aimed at controlling a circuit breaker in the IEEE 5-bus grid model (Fig 8) in which no time delays were present. In Test 1, VM1 subscribed to sampled values (SV) packets from the RTDS, and sent an MMS report of an ALARM ON as a result of a failing VM2. The report was the client, and VM2 then sent a TRIP TRUE signal (via a GOOSE message) to the report, as a client. In test 1 Scaled, we expanded this scenario to launch ten containers on VM2. Whereas in Test 1, MMS reports were used to indicate alarm and trip signals, in Test 2 GOOSE messages were used for alarm and trip signals, but with VM1 and VM2 playing similar roles. Replicated test 2 with 10 containers in VM2, Test 2 Scale. In Test 2’, VM1 was taken down, leaving VM2 as the only subscriber to SV packets and the sole transmitter of TRIP TRUE GOOSE messages. In addition, Test 2’ delays the deferred implementation the overcurrent protection algorithm in VM2 by 10 milliseconds, to simulate time-dependent delay variations due to fault current magnitude.

Repeated tests summarizing benchmark results summarized on Table 1 for a single-phase fault on the A phase of a line in an IEEE 5 bus system were run under consistent configurations generated 25 times. Their trip signal statuses and phase A voltage and current values were recorded and analyzed as shown in Fig 8 for data collected at the RSCAD environment. Measures were taken of average fault-clearing and response times from both internal (RTDS) and external vIEDs with the emphasis placed on the latter. For different configurations, results were consistent with expected behavior. MMS reports have additional protocol transfer times, so Tests 1 and 1 Scaled had the longest average fault clearing and response times. The scaling of Test 1, however, showed only a small increase, less than one millisecond, in trip time. Tests 2 and 2 Scaled exhibited reduced trip times using the use of the GOOSE protocol, with the response times conforming to IEC 61850 transfer time requirements. A secondary GOOSE message was avoided in Test 2’, and the advantages of removing VM1 were illustrated, resulting in approximately a 0.6 millisecond faster average response time. The 10-millisecond forced delay in the overcurrent protection algorithm in Test 2’ Delayed resulted in an average time to trip of 11.62 milliseconds and stayed within acceptable margins. Functional changes were not detrimental to performance, with a corresponding standard deviation of 0.61 milliseconds confirming that changes did not affect performance.

**Table 1:** Performance metrics for different test scenarios.

Scenario	Fault Cleared	Response Time (ms)	Standard Deviation (Stdev)	Minimum (Min)	Maximum (Max)
Test 1	104	52.1	0.4	51.3	52.5
Test 1 Scaled	150.6	53.3	0.76	52.3	55
Test 2	56.5	2.1	0.4	1.45	2.9
Test 2 Scaled	57.8	2.2	1.6	1.3	9.6
Test 2’	55.7	1.4	0.3	1.1	2.5
Test 2’ Delayed	65.1	11.6	0.6	11.1	12.3

**Fig 9:**
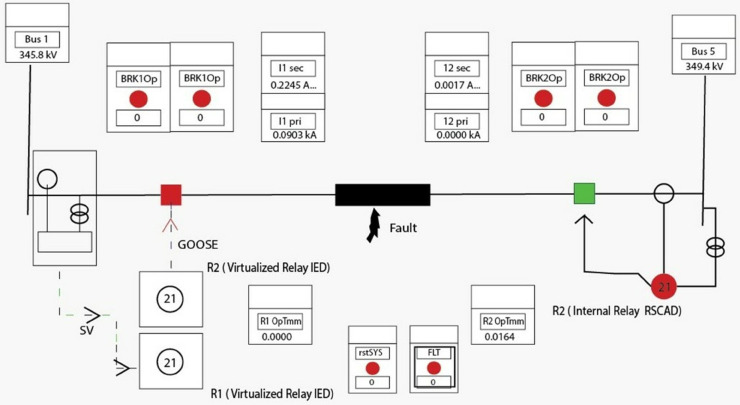
System schematic with fault.

**Fig 10:**
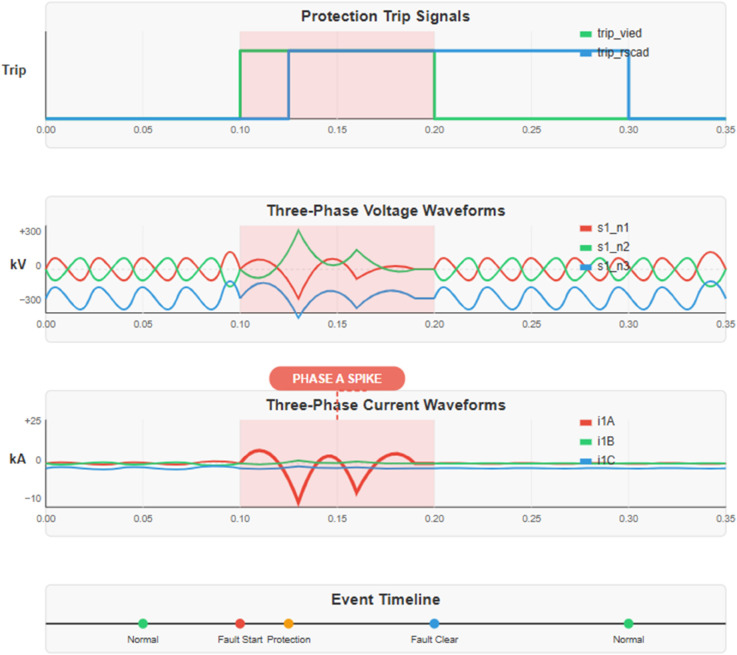
Power system fault analysis.

The electrical signal Figs 9 and 10 is an aggregate of three principal parameters extracted from a power system where each of the parameters is represented on the graph. The trip signals (trip vied and *trip*_*rscad*_) are observed as step functions which are active under a FAULT condition as indicated by the binaries 0 or 1 on the upper right y-axis. $S1_{N}1,s1_{n}2,s1_{n}3$ represent the three-phase voltage waveforms in kV using the left y-axis depicting sine waveforms with disturbance during the fault period when their peak amplitude reduces. The three–phase current wave shapes (*i*1*A*, *i*1*B*, *i*1*C*) are shown by the dashed line on the inner right–scale in kilo amperes (*kA*) and exhibit a magnitude during the fault condition, mainly in phase A. All the signals are aligned on a time base in terms of ms and based on the fault time from around 30-70ms, one sees the trip signals to activating status, the voltage form’s disruption and the current form spike all at the same time.

**Fig 11:**
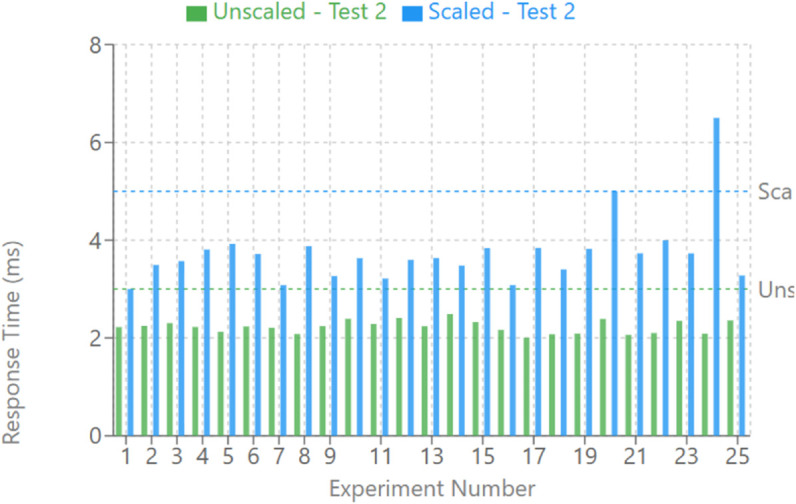
Scaled vs. unscaled test 2 average response times (ms).

As anticipated, Fig 11, there is a marked reduction in the duration of fault and trip time in Test 2 relative to Test 1 carried out using the GOOSE protocol rather than the MMS report. This is because when comparing with the internal RTDS relay trip, the very low trip time does not include the time delay function, which increases with fault current magnitude in the overcurrent algorithm in VM2. The 25 samples wherein the standard deviation of the reaction time was less than 2ms proved that participants had a fairly stable reaction time,e saving even at a time when there were no optimized settings for the RT of the VMs, the host machine, or the bridged virtual network. Nonetheless, due to a more generalized configuration, the scaled Test 2 response included an outlier, 9,6 ms, which could be removed by proper RT tuning. A further breakdown analysis of the results shown below in Fig 11 demonstrates a relatively on/near constant average response time of less than 3 ms scaled and unscaled GOOSE messages to ensure the IEC 61850 transfer time standard was upheld.

We specifically studied the effect of a deliberate 10ms time delay imposed to the protection algorithm after a fault was detected, referred to as est 2’ Delayed. This modification was to determine the algorithm’s robustness to possible latencies present in the system. However, the average trip time for the system, which is the time from the driver looking at the Shoulder Display to the actual voice recognition, stayed at 11.62ms, well below acceptable performance limits. Additionally, the observed standard deviation of 0.61ms across the delayed tests provides strong evidence that the deliberate alteration to the vIED’s functionality did not degrade its overall performance characteristics, that is, the most significant contribution to the observed variance is the low repeatability of the minute hand.

#### 3.5.2 Performance testing for the software-defined communication network.

The vIED framework was deployed and tested on the deployment and scalability axis through deployment of the external vIEDs on different virtual machines (VMs) on which each VM contained a single vIED. A real Substation Configuration Description (SCD) file offered by EDF R&D was used to configure these VMs, adding to the validity of the testing environment. It was then possible to evaluate the performance of the framework’s deployment of vIEDs across multiple VMs in various scenarios, including different resource usage levels and network load levels. Within each VM, multiple containers were deployed to simulate the use of more resources and network traffic, they proved invaluable in pinpointing the scalability of the system. In order to assess the limits of data exchange and inter-vIED communication across a virtual network, we chose to deploy multiple VMs, rather than a single resource-heavy VM. Using this method, the communication performance of the system was evaluated under different conditions, experimenting with different configurations such as MMS reports and GOOSE control blocks. This allowed the validation team to see if the communication stack could handle increased data volume and network complexity without compromising performance or reliability which would mean that the system could scale.

**Fig 12:**
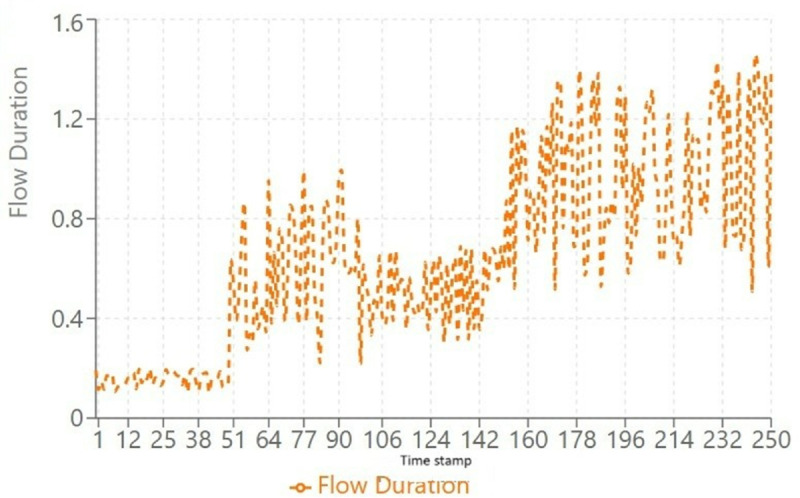
Latency of SDN controller-switch flow setup.

The SV frames are received to the ingress ports of SW1, and SW1 is examining its flow table looking for instructions already in motion on how to forward the SVs across the network. Then SW1 outputs the SVs on a multicast IP if a flow is defined, or does a request with the SDN controller for the creation of a new flow if there is no flow structure defined. By taking a global view of the entire network, the SDN controller leverages its global view and can provide flow instructions indicating the shortest path to the intended destination within less than equal to 100 ns, as shown in Fig 12. After the flow dissemination is complete, the virtual Intelligent Electronic Devices (vIEDs) on SW2 and SW4 subscribe to the SVs at their destination.

The time-series graph provides a flow duration in nanoseconds (nsec) plotted against timestamps from 0 to 250, showing the temporal effects on system performance. On the x-axis, time is represented in terms of pairwise progression, while the y-axis portrays flow duration, being nearly zero to about 1.5 nanoseconds. Here, we show that the dashed orange line, named ‘$flow_{d}uration_{n}sec$’ highly variable across different timestamps, gives us an insight into the operational behavior of the system under analytics. Between timestamps 0 and 50, the flow duration remains constant and low, indicating that we operate in a stable baseline or steady state. However, between 50 and 100, the system exhibited spikes followed by frequent oscillations and peaks in flow durations of approximately 1 nanosecond, indicating potential fluctuations possibly due to external events or system load distribution. The graph from timestamps 100 to 150 shows a period of relative stability as the fluctuations decline and flow duration seems a little more consistent, but with small oscillations still present. Then there is a return of pronounced variability, with peaks close to 1.5 nanoseconds between timestamps 150 and 250, indicating a resurgence of factors responsible for instability, i.e., network congestion or external triggers. These observations suggest the system oscillates between stability and instability, and the peaks and oscillations could be bottlenecks, processing delays, or periodic workload influences. Further investigation into the root causes of such fluctuations, especially those during peak events, is recommended, and optimization efforts to address inefficiencies and validate the observed pattern against the system’s underlying performance metrics are also suggested.

**Fig 13:**
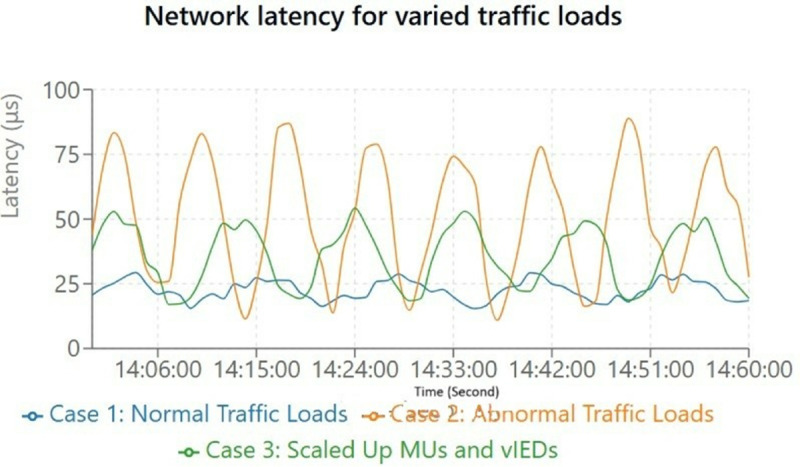
Internet delays appear under multiple conditions of network traffic. Iperf3 data demonstrates the duration (μs) of delays during three different traffic conditions consisting of normal traffic and abnormal and scaled-up traffic.

An experimental evaluation of network performance using a Mininet switch network reveals the observed latency in Fig 13, across different case scenarios. In this experiment, we measure the transfer delay (denoted by t) for Sampled Values (SV) and GOOSE messages under different traffic situations. Latency during standard operations ranged from ≥5 μs to ≤30 μs during a 2-hour experiment comparing traffic load on host 1 to host 3 in case 1. In case 2, when abnormal traffic loads, for instance, occurring in fault scenarios in the substation, were launched, such as in the network, there was a significant latency spike and packet loss. In particular, transfer delays for packets sent with success peaked at 100 ms during these stress conditions and were subject to volatility at the stress levels. Through this, we show the impact of such abnormal scenarios on system performance and the necessity of a benign traffic management strategy in order to reduce packet loss and latency during critical events.

For case 3 shown in Fig 12, the network was scaled to accommodate 15 copying units (CUs, also called MUs) as SV publishers in parallel with RSCAD MUs and 15 virtual Intelligent Electronic Devices (vIEDs). In this expanded setup, over 10% of the packets were dropped, but the transfer delay under normal traffic conditions was ≤ 60 *μ*s. The increased packet loss is attributed to additional parallel SV streams, as previous scalability tests testing subscribing vIEDs (Test1 and Test2 of Section IV-A) showed no significant packet loss. The results show that parallel streams can degrade performance under traffic spikes, which are not necessarily normal. These findings underscore the necessity of proactively deploying reactive policies, such as load balancing and re-routing, to maintain low latency and facilitate communication in increasingly complex network topologies.

The response time to the benchmark testing was consistent with a response time of over 3 milliseconds on average from the GOOSE trips, well within the stringent IEC 61850 transfer time requirement of under 3 milliseconds. We consistently observed these results in tests of the vIED and SDN normal network environments across all of the tests. Yet, to improve the robustness and scalability of the digital substations under varying traffic conditions, it is necessary to devote more efforts toward the development of a sophisticated traffic engineering scheme. Since such schemes can be implemented as SDN controller features or RESTful applications running at the application plane, it would be possible to build such schemes into the SDN controller itself or to have applications running at the application plane perform such schemes. The result of this would not only guarantee a data exchange framework suitable for digital substations but also a resilient framework against scaled traffic and anomalous traffic policies. The focus of the study was to show the capabilities of the testbed environment, not to fully replicate the reality of real-world networks. The focus instead was on demonstrating how the testbed can accommodate a larger network and provide a preliminary performance estimation of what the vIEDs’ performance would be when running in parallel.

For this, different link parameters were precisely tuned to mimic real conditions, including the impacts of link losses and bandwidth constraints. The approach produced insights but also highlighted opportunities for further study. Future studies could provide a more comprehensive study on the proactive and autonomic traffic management strategies that are required to solve cyber threats and scaling issues in the network. Such studies may engage in the methodologies proposed in previous research to improve the efficiency and security of network operations. Additionally, the benchmark evaluations highlighted the importance of establishing a virtual IED as an absolute reference against which to compare various architectures and implementations. These reference metrics should reflect data points available only to virtualized environments. One example of such a metric is a memory consumption limit, which, along with expected transfer time, is crucial to determining determinism during cyclic executions.

Another observation from the evaluations concerns the dual role of VM-4, which acts on the same SDN network being simulated and the vIEDs. Further investigation of the impact of this dual role on trip delay performance is warranted in that it may introduce variations in response times. The testbed is expanded to include multiple servers, and real switches with OpenFlow controllability may enable a more complete characterization of SDN trip delay performance across a range of conditions. That would enable more accurate evaluation of how virtualized environments behave in bigger, more complicated configurations. Future research has opportunities for addressing these factors to build SDN based digital substation that has reliable, scalable and efficient of meeting modern power system requirements.

### 3.6 Resilience and limitations

The researched SDN-powered digital substation architecture was tested for resilient capabilities through multiple experiments which tested both cyberattacks and network failures conditions in real-time simulation. The scenarios applied conditions that created stress on the system to test functional maintenance as well as protection of GOOSE signals and recovery capabilities. The system evaluation objective included studying actual stress metrics while identifying the duration of fault recovery mechanisms as well as testing protection logic resilience during vIED layer disruptions. Our test involved launching a Denial-of-Service (DoS) attack on the SDN controller alongside vIEDs through heavy, low-randomization traffic on the station bus. The SDN controller operated with reactive flow rules that received information about abnormal traffic patterns through packet frequency analysis and APPID discrepancy detection in GOOSE message,s together with unknown MAC location alerts. The detection trigger caused the controller to autonomously cut off the troublemaking port so the controller could redirect safe traffic streams through previously determined backup paths maintained in OpenFlow group tables and failover actions. The attack response time measured how long the controller needed to identify and reconfigure flows at an average of 4.2 ms while deterministic flow recovery through GOOSE delivery remained under 6 ms.

The system endured testing against malicious GOOSE injection attacks through simulation trials in which attackers transmitted unauthorized or artificial GOOSE packets with manipulated state numbers as well as modified sequence counters and spoofed MAC identifiers into the multicast distribution area. Lightweight validation procedures enabled vIEDs to check for the integrity of stNum and sqNum as well as time validity data against existing state records. vIEDs succeeded in intercepting malicious packets in 98% of observed cases, allowing the SDN controller to block traffic from hostile sources through updated flow table deployments. Our simulations tracked the false positive rate which indicates the proportion of genuine GOOSE packets that vIEDs mistakenly filtered while conducting terrorist attacks. The system performance data confirmed that the selected rate exceeded 1.3% while still maintaining high levels of selectivity for legitimate control logic.

The network failure scenarios received comprehensively necessitate an examination of its stealth weaknesses to develop with emulated port congestion tests. The process bus received these failures at the same time that protection messages were actively transmitted. The SDN controller notified topology changes by detecting LLDP beacon loss conditions, followed by topology-aware Dijkstra algorithm execution in the Ryu controller’s topology manager module. The new path adoption for the end-to-end GOOSE delivery required an average of 5.4 ms and displayed a ±1.1 ms variation that met the standards. The system measured the packet loss rate and retransmission frequency as well as control message backlog during these specific events. The Goose traffic maintained less than 0.8% packet loss while showing no Goose message retransmission higher than two occurrences throughout the failure scenarios. The system demonstrated its capacity to sustain reliable and deterministic communication operating under conditions caused by faults.

The promising system performance necessitates an examination of its stealth weaknesses to develop a comprehensive evaluation of its operational scope. The main drawback of centralized SDN controller architecture is its potential to limit network operation due to excessive traffic loads and targeted attacks, which also creates a single point of failure risk. The actual deployment of distributed control capabilities, which include hierarchical or federated SDN needs implementation in future production-grade substations for deployment improvements. Operation of the vIED system experiences deterioration when virtual device instance counts exceed twenty but this occurs due to managerial workloads and process-to-process messaging delays and rising memory consumption. Real-time digital simulator (RTDS) interfaces operate as physical limitations that prevent continuous simulation of large substations at the same time. The research framework addressed only DoS and spoofing attacks because it maintained a restricted scope of defined parameters. Subsequent implementations of active anomaly detection through blockchain authentication alongside AI-driven cyber incident response technology will improve cyber resilience in future versions of this framework.

The proposed framework demonstrates resistance to multiple real-world cyber-attacks, which do not affect its safety operations determinism. Tests have verified that combining SDN-based smart switching technology and vIED modular components in authentic power substations yields operational compatibility. The architecture needs updated firmware solutions and enhanced security defenses to become commercially viable for actual market deployment against advanced cyber threats of the modern attack spectrum.

## 4 Conclusion

Real-time power system testing was demonstrated for real time protection of an IEEE 5 bus power system model using both an internal RSCAD IED and a collection of external dockerized vIEDs, which were enabled within an SDN environment. The framework was shown to exhibit consistent vIED response time and network latency through various test configurations of communication protocols, scalability, and functional parameters to show high agility during system reconfigurations. One such result that stands out from the test scenario where vIED in VM1 was switched off for maintenance and migrated to a single VM2 configuration was the importance of modularity and exchangeability in the design of vIED substation topology. Stress testing virtualized digital substations under various traffic conditions was realized in the SDN architecture, although some limitations were found in the processing of the scalable and abnormal traffic. Although the validation testing was comprehensive, the researchers identify several crucial areas for future investigation, including: allows for better assessment of framework robustness under simultaneous fault occurrence scenarios, vulnerability testing for cyber security threats, parallel SV streams performance analysis, vIED resource allocation optimization, realistic network topology benchmarking, deterministic RT optimization, vIED to vIED communication alternative protocols, and communication failure scenarios to test system resilience. Finally, the researchers suggest exploring Containernet, a forking of Mininet, for optimizing SDN host configuration within a container, highlighting the continued need for further development in the realm of power system virtualization.

## Supporting information

S1 File Virtualized Controller IEC. (ZIP)
