# Supplementary figures and images for: Agile cyber defense: Enhancing digital substation resilience with SDN-based smart switching
Source: PLoS One. 2025 Nov 18;20(11):e0330521. doi: 10.1371/journal.pone.0330521 (PMC12626286; doi:10.1371/journal.pone.0330521)

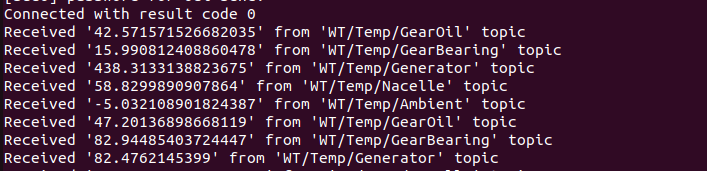

Supplement: S1 File — (ZIP) [file pone.0330521.s001.zip › Virtualized Controller Implementation and Testing for Software-Defined IEC 61850/Dependability_Assessment/ECP_Node/Results/Screenshot from 2023-12-09 17-36-12.png]

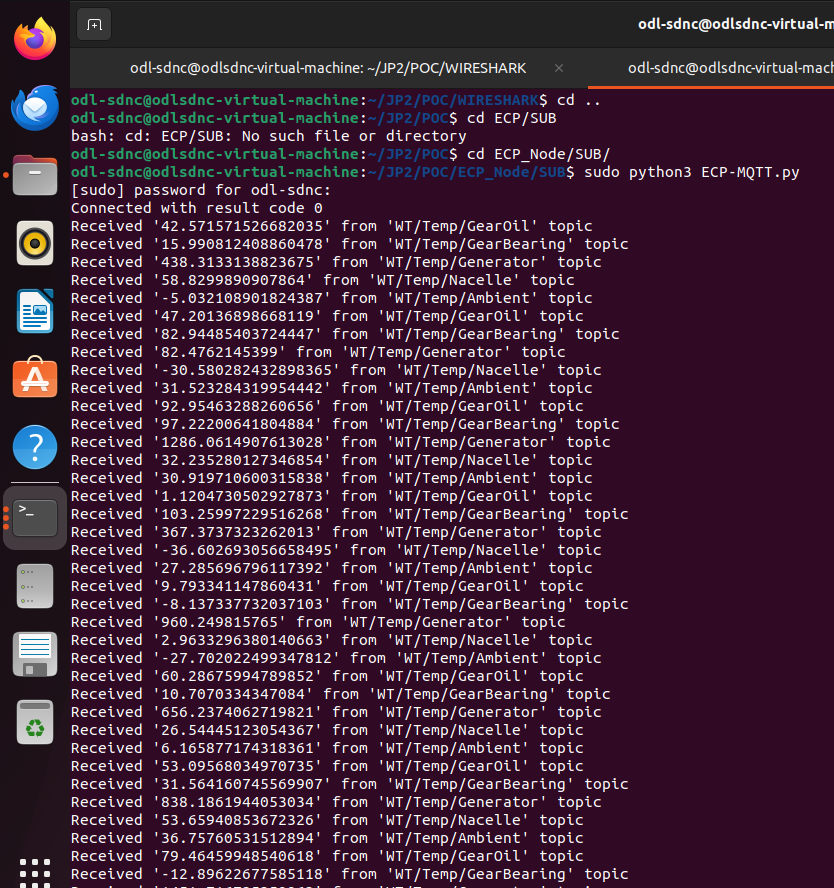

Supplement: S1 File — (ZIP) [file pone.0330521.s001.zip › Virtualized Controller Implementation and Testing for Software-Defined IEC 61850/Dependability_Assessment/ECP_Node/Results/Screenshot from 2023-12-09 17-35-38.png]

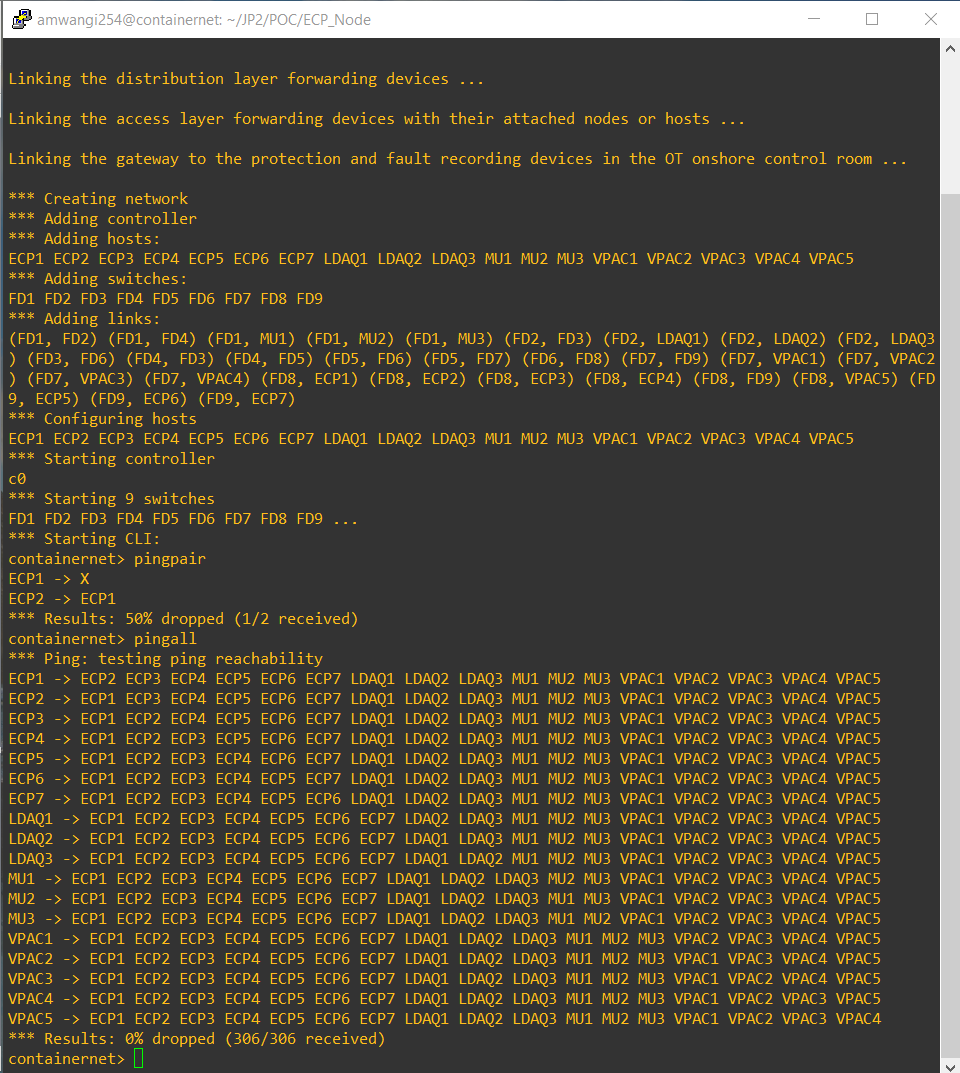

Supplement: S1 File — (ZIP) [file pone.0330521.s001.zip › Virtualized Controller Implementation and Testing for Software-Defined IEC 61850/Dependability_Assessment/ECP_Node/Results/Ping_Results.PNG]

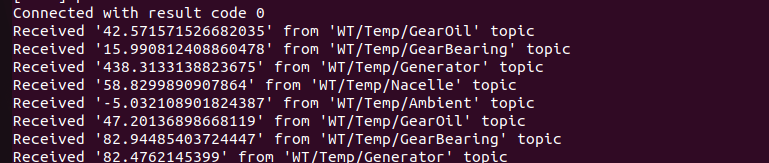

Supplement: S1 File — (ZIP) [file pone.0330521.s001.zip › Virtualized Controller Implementation and Testing for Software-Defined IEC 61850/Dependability_Assessment/ECP_Node/Results/ECP_MQTT.png]

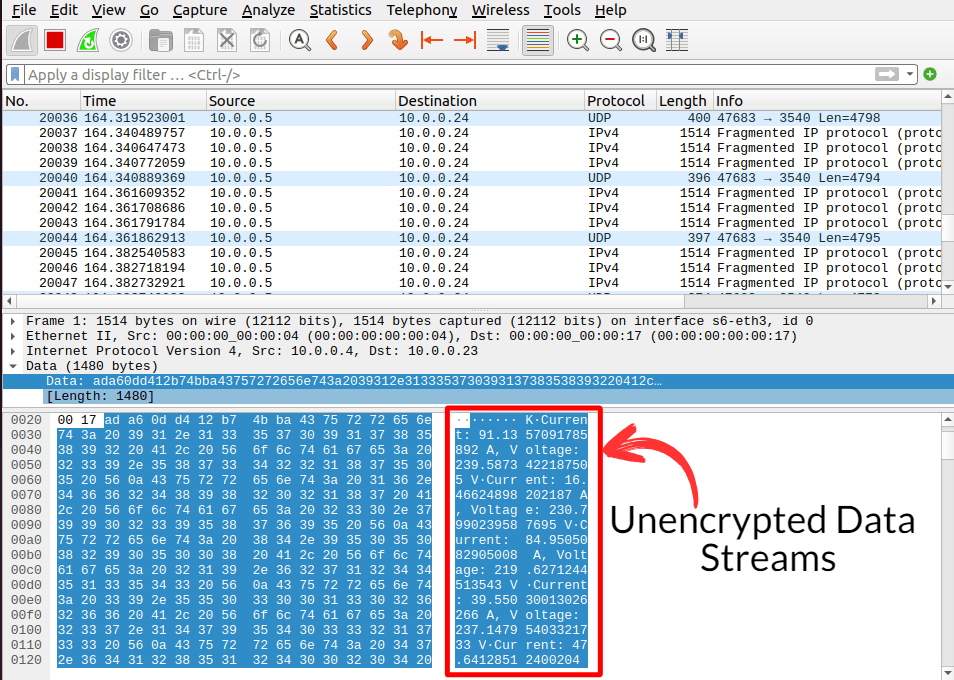

Supplement: S1 File — (ZIP) [file pone.0330521.s001.zip › Virtualized Controller Implementation and Testing for Software-Defined IEC 61850/Dependability_Assessment/ECP_Node/Results/unencrypted.jpeg]

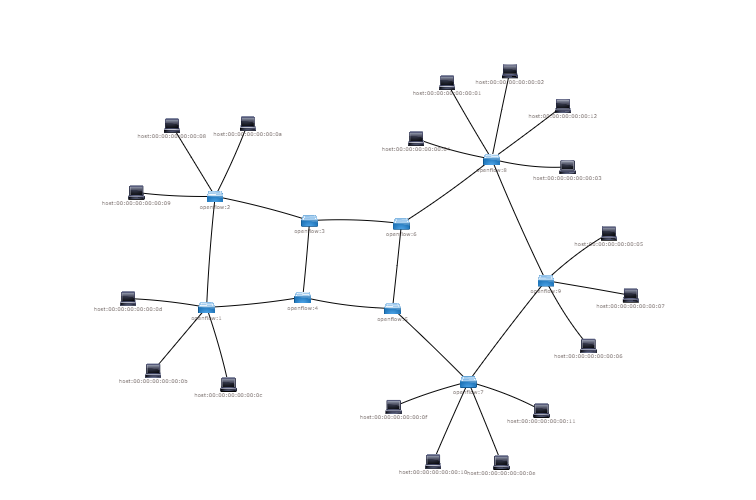

Supplement: S1 File — (ZIP) [file pone.0330521.s001.zip › Virtualized Controller Implementation and Testing for Software-Defined IEC 61850/Dependability_Assessment/ECP_Node/Results/Network Topology.png]
